# Seed Transmission of Cowpea Mild Mottle Virus in Common Beans in Brazil

**DOI:** 10.3390/v18070752

**Published:** 2026-07-07

**Authors:** Bruna Pinheiro-Lima, Andreza H. Vidal, Gustavo P. Felix, Dione M. T. Alves-Freitas, Cristiano Lacorte, Josias C. Faria, Emanuel F. M. Abreu, Patricia Valle Pinheiro, Fernando L. Melo, Simone G. Ribeiro

**Affiliations:** 1 Embrapa Recursos Genéticos e Biotecnologia, Brasília 70770-917, Brazil; 2 PPG BioMol, Universidade de Brasília, Brasília 70275-970, Brazil; 3 Embrapa Arroz e Feijão, Santo Antônio de Goiás 75375-000, Brazil

**Keywords:** seed-transmitted, CPMMV, carlavirus, *Phaseolus vulgaris*

## Abstract

Cowpea mild mottle virus (CPMMV) is a carlavirus that is transmitted by whiteflies and can also be spread through seeds in cowpea, soybean, and common bean. Seed transmission of CPMMV has been described in various countries; however, it was only recently reported in Brazil as a seed-transmitted virus in soybean. CPMMV has spread widely in bean-growing areas in recent years. Most Brazilian farmers use harvested grains as seed for reseeding, increasing the risk of seedborne infection. In this study, we examine seed transmission of CPMMV in two bean cultivars using RT-PCR in combination with nucleic acid hybridization. The plants evaluated were obtained from seeds harvested in commercial and experimental fields and from seeds collected in transmission experiments using either mechanical or whitefly inoculation. The observed seed transmission was estimated between 10 to 45% for ‘BRS FC 401 RMD’ and 13 to 22% for ‘Pérola’. In addition, evidence of secondary transmission (23.3%) by *Bemisia tabaci* MEAM1 was observed. These results suggest that CPMMV could be spread in bean fields by sowing infected seeds and that germinated plants could serve as an inoculum for whitefly transmission. This is the first report of seed transmission of CPMMV in common bean in Brazil.

## 1. Introduction

Common bean (*Phaseolus vulgaris*) is an essential food crop in Brazil, with an annual production of approximately 3.3 million tons [1]. Viral diseases significantly limit bean yield, with the most prevalent virus being bean golden mosaic virus (BGMV; *Begomovirus costai*), which belongs to the family *Geminiviridae* [2]. In the last decade, cowpea mild mottle virus (CPMMV, *Carlavirus vignae*), a member of the family *Betaflexiviridae,* has also been identified as a concern [3]. Both viruses have been found at very high incidences and often occur in mixed infections alongside the whitefly *Bemisia tabaci*-transmitted bean-associated cytorhabdovirus (BaCV, *Betacytorhabdovirus caricae*), which belongs to the *Rhabdoviridae* [4].

CPMMV has a single positive-sense RNA of approximately 8.2 kb in length, presenting a cap structure at the 5′ end and a poly-A tail at the 3′ end. The CPMMV genome consists of six ORFs: ORF1 encodes a replicase with four conserved domains (methyltransferase, C23 peptidase, RNA helicase, and RNA-dependent RNA polymerase); ORFs 2, 3, and 4 encompass the triple gene block; ORF 5 encodes the coat protein; and ORF 6 codes for a nucleic acid-binding protein [5,6]. Viral particles have flexuous filamentous shape, measuring 13 × 650 nm, and are usually found in aggregates within the cytoplasm of palisade parenchyma and mesophyll cells [7].

Virus transmission occurs through the whitefly vector in a non-circulative manner and via experimental sap transmission. CPMMV infection can induce a range of symptoms depending on the host species and genotype. When the virus was first detected in cowpea (*Vigna unguiculata*) in 1973, some cowpea genotypes showed systemic chlorotic mottling while others were asymptomatic [8]. CPMMV was first reported in Brazil in 1983, causing leaf distortion and mosaic in common beans [9]. However, it was only in the 2000s that CPMMV became significant in Brazil as a cause of stem necrosis and stunting in soybeans (*Glicyne max*) [10].

Seed transmission is a primary driver of plant virus epidemics. By facilitating viral survival between seasons and long-distance geographic dissemination via global trade, seed-borne infections establish early primary inoculum sources. Even at low transmission rates, these infected seedlings trigger widespread outbreaks when amplified by vector activity. CPMMV seed transmission has been tested using different virus isolate/host plant combinations, yielding variable results. Seed transmission was reported for the Ghana isolate in cowpea, soybean, and common bean, with rates ranging from 2% to 91% depending on the plant species. In soybean, seed transmission was very high (91%), whereas transmission rates were much lower (2%, 4%, and 13%, respectively) in three *P. vulgaris* cultivars (Canadian Wonder, The Prince, and Comtesse de Chambord). Moreover, all plantlets evaluated were asymptomatic [8]. Seed transmission of the CPMMV Venezuelan isolate was suspected due to the presence of symptoms in 40% of the yardlong bean (*V. unguiculata* subsp. *sesquipedalis*) seedlings evaluated, but no other diagnosis approach was used to confirm virus infection [11]. In India, seed transmission was reported for several soybean cultivars, with rates ranging from approximately 0.60% to 14% [12]. Another study confirmed CPMMV seed transmission in soybeans, with higher transmission rates (75% in the F1 generation and 100% in the F2) and in cowpea [13]. In Thailand, a frequency of nearly 1% was observed in soybeans [14]. In Indonesia, CPMMV was not detected in soybean seeds by ELISA [15]; however, CPMMV seed transmission was later detected in several soybean varieties using a dot immunobinding assay [16]. The latter findings indicated that seed transmission of CPMMV is dependent on the soybean cultivar [16]. Additionally, CPMMV seed transmission has been reported in cowpeas in Nigeria and Uganda [17,18].

In Brazil, seed transmission of CPMMV in soybeans and common beans has been investigated in several instances by visualizing symptoms in potentially vertically infected seedlings, with negative results [9,10,19]. However, visual inspection is not the most sensitive method for determining the incidence of seed transmission of viral pathogens, as vertically infected seedlings often show few or no symptoms [20]. Recent studies indicate that more sensitive methods may be necessary to detect seed transmission of CPMMV, especially when seed tissue samples are analyzed [13]. Silva et al. [21] provided evidence of a low level (0.375%) of seed transmission of CPMMV in the soybean cultivar BMX POTÊNCIA RR. Taken together, these findings suggest that seed transmission of Brazilian CPMMV isolates occurs in at least one soybean cultivar; however, in common beans, it remains an important unresolved issue.

Certified bean seeds account for less than 20% of the total planted area in Brazil, with most farmers replanting seeds (i.e., grain) saved from previous harvests [22]. This practice increases the risk of seed-transmitted diseases, a concern associated with the biosecurity of plant viruses. Even low infection rates through seeds, when combined with secondary spread by whiteflies, can lead to severe outbreaks [20]. The presence of CPMMV in young bean plants—observed even in the absence of whiteflies in the field (Pinheiro-Lima, personal communication)—indicated that some transmission through seeds could be occurring. In this study, we examined the seed transmission of CPMMV in two distinct bean cultivars. To ensure a high sensitivity in detection, we assessed CPMMV infection by RT-PCR and nucleic acid hybridization techniques. We also analyzed whether plants grown from infected seeds function as an inoculum source for whitefly transmission.

## 2. Materials and Methods

### 2.1. Seeds and Plant Material

Transmission experiments were carried out between 2017 and 2020 using two common bean cultivars: ‘BRS FC 401 RMD’ and ‘Pérola’. The bean seeds were sourced from five different origins: (i) Healthy ‘BRS FC 401 RMD’ and ‘Pérola’ bean plants were cultivated for several generations in an insect-proof greenhouse and sprayed periodically with insecticide (cyantraniliprole, 0.25 mL/L) at Embrapa Arroz e Feijão, Santo Antônio de Goiás, GO, Brazil. Plants grown under these conditions never showed virus symptoms and consistently tested negative for viruses. Therefore, seeds collected from these plants were considered free of virus. (ii) In 2017, seeds were collected from potentially CPMMV-infected ‘BRS FC 401 RMD’ plants situated in a field at Embrapa Arroz e Feijão. At the time of collection, these plants were at the R9 phenological stage [23], with nearly all pods and leaves already dried. In earlier stages, these plants presented virus-like symptoms similar to those described by Alves-Freitas et al. [24], with an incidence exceeding 80%. (iii) Similarly, in 2016, seeds from the ‘Pérola’ bean plants were harvested from a commercial farm in Cristalina, GO. Plants were at R9 phenological stage, and the farmer reported nearly 100% virus-like symptoms in the field earlier in the season. (iv) ‘BRS FC 401 RMD’ seeds were collected from plants infected by CPMMV (and BaCV), which had been inoculated with whiteflies in previous experiments documented by Pinheiro-Lima et al. [4]. (v) ‘BRS FC 401 RMD’ and ‘Pérola’ seeds were obtained from bean plants experimentally infected with CPMMV through mechanical inoculation. Healthy seeds of both cultivars were cultivated inside an insect-proof cage in a greenhouse at ambient conditions. Infected (CPMMV, BaCV, and BGMV) leaves from a bean plant collected in 2016 in Cristalina, GO [4], were macerated with inoculation buffer [0.01 M potassium phosphate buffer (Sigma-Aldrich, St. Louis, MO, USA), pH 8.0; containing 0.25 M EDTA (Sigma-Aldrich, St. Louis, MO, USA) and 0.01% sodium sulfite (Sigma-Aldrich, St. Louis, MO, USA)]. The inoculum was then rubbed onto the leaves of 10 plantlets of each cultivar, which had been previously dusted with carborundum (600 mesh, Sigma-Aldrich, St. Louis, MO, USA). Thirty days post-inoculation (dpi), the plants were tested for CPMMV infection by RT-PCR. Infected plants were further cultivated until maturity, and their seeds were collected. All seeds were air-dried and stored in paper bags at 4 °C for up to 3 years.

### 2.2. Seed-Transmission Experiments

**Figure 1:**
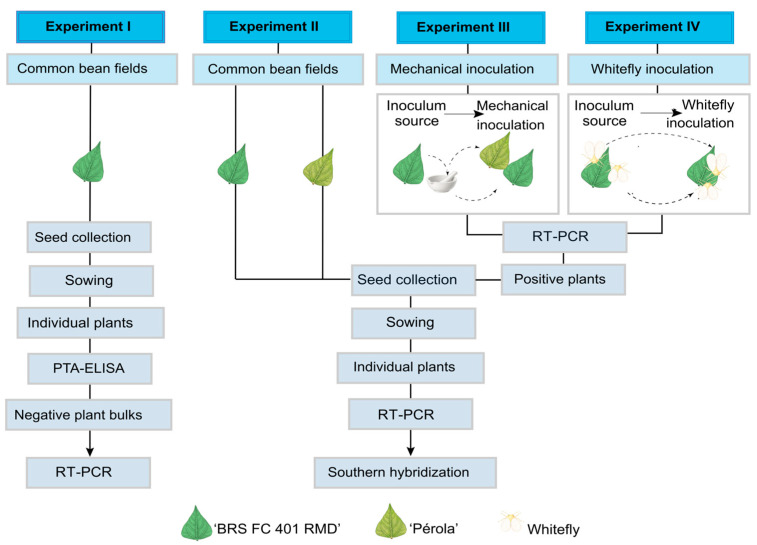
Experiments conducted to evaluate the seed transmission of CPMMV in ‘BRS FC 401 RMD’ and ‘Pérola’ common bean cultivars in Brazil. The seeds used in the grow-out tests were collected from common bean plants in an experimental field (Experiments I and II) and from a commercial field with high viral incidence (Experiment II). Additionally, seeds were collected from mechanically inoculated plants (Experiment III) and from plants inoculated with whiteflies (*Bemisia tabaci* MEAM1) (Experiment IV). In Experiment I, 190 plants were individually tested for CPMMV by PTA-ELISA. Subsequently, 19 bulks, each comprising 10 plants, were formed and tested by RT-PCR. In the other experiments, all plants were individually tested using RT-PCR and nucleic acid hybridization. Created with Inkscape 1.4.2.

A series of four experiments was conducted to assess the transmission of CPMMV by the seeds of common beans ‘BRS FC 401 RMD’ and ‘Pérola’ (Figure 1). Seeds from both naturally infected and experimentally inoculated plants were utilized. All assays were based on grow-out tests, in which plants originating from potentially infected seeds were screened for virus infection.

The first experiment (Experiment I, Figure 1) was carried out at Embrapa Arroz e Feijão, Santo Antônio de Goiás, Brazil. Seeds of the cultivar ‘BRS FC 401 RMD’, obtained from a field with a high incidence of CPMMV, were used for the transmission tests. Seeds were individually sown and maintained in a greenhouse free of whiteflies, with precautions taken to avoid insect transmission. This included proper greenhouse insulation and weekly application of cyantraniliprole (0.25 mL/L) (Benevia^®^, FMC Agricola, Campinas, Brazil). Twenty days after germination (dag), plants were examined for visual symptoms, and leaf tissue was collected for virus detection. Individual samples were collected for PTA-ELISA, and negative samples were subsequently analyzed in bulk using RT-PCR.

The other three experiments were conducted in a growth chamber at 24 °C (±4 °C) with a 12 h photoperiod at Embrapa Recursos Genéticos e Biotecnologia, Brasília, Brazil.

In Experiment II (Figure 1), the objective was to investigate the occurrence of CPMMV transmission via seeds collected from naturally infected plants in the field. For this purpose, seeds of ‘BRS FC 401 RMD’ from the same lot as described above for Experiment I, along with seeds of ‘Pérola’ collected in Cristalina, Goiás, Brazil, were grown in a mixture (1:1) of autoclaved soil and Plantmax HT substrate (Eucatex Agro, Paulínia, Brazil) in 500 mL pots. The first fully developed leaf was collected at 30 dag, and plants were evaluated for virus presence by RT-PCR, followed by Southern blot hybridization with a probe specific for CPMMV.

Experiments III and IV (Figure 1) assessed CPMMV seed transmission using seeds obtained from experimentally inoculated plants via artificial mechanical inoculation or biological inoculation (by the whitefly vector), respectively. In Experiment III, 24 seeds of ‘BRS FC 401 RMD’ and 25 of ‘Pérola’ were sown in 500 mL pots as described in Experiment II. Similarly, in Experiment IV, 25 ‘BRS FC 401 RMD’ seeds were sown. Plants were tested for CPMMV infection at 30 dag using RT-PCR followed by Southern blot hybridization assays with a probe specific for CPMMV.

In previous studies, BaCV was found at high incidence in mixed infections with CPMMV in bean fields in Santo Antônio de Goiás and Cristalina, and was transmitted by *B. tabaci* to both ‘BRS FC 401 RMD’ and ‘Pérola’ [4,24]. Therefore, the plants grown from seeds in Experiment II and IV were also tested for BaCV by RT-PCR followed by Southern blot hybridization with a specific BaCV probe.

### 2.3. Indirect ELISA

Samples from 190 plants of the ‘BRS FC 401 RMD’ variety, grown from field-collected seeds, were tested for CPMMV infection using the indirect or plate-trapped antigen PTA-ELISA method. Each sample consisted of three 1 cm leaf discs taken from each plant. The CPMMV antibody used in the analysis was kindly provided by Dr. Álvaro M. R. Almeida from Embrapa Soja, Londrina, Brazil. The PTA-ELISA method was performed according to the protocol by Arias et al. [25]. Each sample was analyzed in triplicate, and control samples included both negative controls from healthy bean plants and positive controls from known CPMMV-infected plants.

### 2.4. RNA Extraction from Bean Leaves

For Experiment I (Figure 1), samples were collected in 19 bulks of ten plants, consisting of one leaf disc from each ‘BRS FC 401 RMD’ plant, totaling approximately 100 mg for each bulk. Total RNA was prepared using RNeasy Plant Mini Kit (Qiagen, Germantown, MD, USA) according to the manufacturer’s instructions. For subsequent experiments, total RNA was extracted from 100 mg of bean leaves from individual plants using TRIzol Reagent (Invitrogen, Carlsbad, CA, USA), according to the manufacturer’s protocol. The quality and quantity of RNA preparations were assessed using a 1% agarose gel and measurements from a Nanodrop spectrophotometer (Thermo Fisher Scientific, Waltham, MA USA).

### 2.5. Virus Detection by RT-PCR

To detect CPMMV in ‘BRS FC 401 RMD’ plants grown from seeds collected from field plants (Experiment I, Figure 1), cDNA was synthesized from 2 µg of RNA, using the GoScript cDNA synthesis kit (Promega, Madison, WI, USA) and random octamer primers, according to the manufacturer’s instructions. All primers used in this study are described in Table S1. PCR reactions were conducted using 1 µL of cDNA, along with CPMMV-specific primers CPMMV-4000F and CPMMV-4500R [3] (Table S1), and temperature cycle conditions as described by Alves-Freitas et al. [24].

For the other experiments, RNA samples were treated with 2 units of TURBO DNase (Life Technologies, Carlsbad, CA, USA) at 37 °C for one hour. Afterward, reverse transcription was carried out using 50 mM oligo dT and 10 mM random primers, 200 U of M-MLV Reverse Transcriptase (Promega, Madison, WI, USA), and 2 µg of RNA. Only cDNAs that were assessed for quality via PCR using specific primers for the actin 11 gene [26] were used. All PCRs were carried out using 1 µL of cDNA, 1 µL each of 10 mM specific primers (qAct11_F and qAct11_R; qCPMMV_4144F and qCPMMV_4339R; BaC_1F and BaC_1579R), 0.8 µL of 10 mM dNTPs, 0.2 µL of (5 U/µL) Taq DNA polymerase (Invitrogen, Carlsbad, CA, USA), 2.5 µL of 10x Taq DNA polymerase buffer, 1 µL of 50 mM MgCl_2_, and 17.5 µL of water. The reactions underwent an initial denaturation step at 95 °C for 3 min, followed by 35 cycles of denaturation at 95 °C for 30 s, annealing at 58 °C for CPMMV or 61 °C for BaCV for 30 s, and extension at 72 °C for 30 s or 1 min and 40 s. PCR products were resolved on a 1.2% agarose gel.

### 2.6. Virus Detection by Nucleic Acid Hybridization

Nucleic acids from the gel were transferred to Hybond XL nylon membranes (GE Healthcare, Chicago, IL, USA) using alkaline transfer, following the manufacturer’s instructions. DNA was fixed to the membranes by UV crosslinking for 30 s using a UV Stratalinker 1800 apparatus (Stratagene, San Diego, CA, USA). The membranes were hybridized with radiolabeled probes specific to CPMMV or BaCV, prepared using the Amersham™ Rediprime™ II DNA Labeling System (GE Healthcare, Chicago, IL, USA) and [α^32^P] dCTP, with the viral genome regions of the corresponding PCR fragments as templates. Signals were detected by exposing the blots to Fujifilm BAS-IP MS imaging plates, and the resulting images were acquired using a Fujifilm FLA-3000 scanner (Fujifilm Corporation, Tokyo, Japan). The Fujifilm FLA-3000 scanner displays highly intense signals as pink pseudocolor pixels. For figure preparation, images were uniformly converted to grayscale to facilitate visualization of hybridization signals; no adjustments affecting the underlying data were made.

### 2.7. Whitefly Transmission of Seed-Transmitted CPMMV

**Figure 2:**
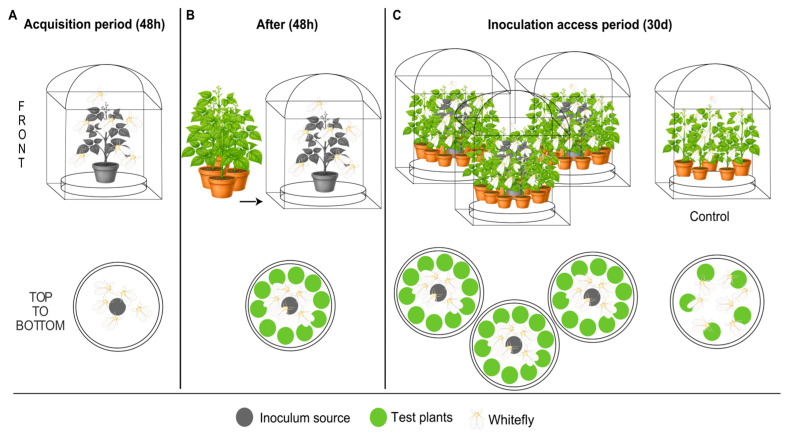
Whitefly-mediated (*Bemisia tabaci* MEAM 1) transmission of seed-transmitted cowpea mild mottle virus (CPMMV). (**A**) Three CPMMV-positive ‘BRS FC 401 RMD’ plants, identified in a seed transmission assay, served as the inoculum source. These plants were exposed to whiteflies during a 48 h acquisition access period. (**B**,**C**) Following this, ten healthy plantlets were introduced into each cage and were maintained for 30 days. At the end of the assay, the younger leaf of each test plant was collected and tested for CPMMV infection. A control experiment was conducted in a separate cage with five healthy bean plantlets and non-viruliferous whiteflies. Created with Inkscape 1.4.2.

A whitefly-mediated transmission assay was conducted to determine if vertically transmitted CPMMV could be transmitted to healthy bean plants by the natural vector (Figure 2). Three CPMMV-infected ‘BRS FC 401 RMD’ plants, which tested positive in Experiment III, served as source plants. These plants were individually placed in three BugDorm cages (MegaView Science Co., Taichung, Taiwan) within a growth chamber maintained at 24 °C (±4 °C) and a 12 h photoperiod. The insect cages were made with anti-aphid mesh and had long sleeves for manipulating the insects inside (without opening the cage door), thereby preventing escapes. Yellow sticky traps were installed in the growth chamber to capture any insects that might escape. *B. tabaci* MEAM1 whiteflies were reared on cabbage plants, which are non-hosts for CPMMV, in a rearing facility at the Universidade de Brasília, Brasilia, Brazil. Approximately 400 whiteflies were collected from the cabbage plants and transferred to each of the three source plants for a 48 h acquisition period. After this period, 10 healthy ‘BRS FC 401 RMD’ test plantlets (at 10 days after sowing) were introduced to each of the three cages to coexist with the insects and the inoculum source for 30 days. A total of 30 test plants were evaluated, arranged in three replicates of 10 plants each. As a control, whiteflies were placed in a separate tent with five healthy bean seedlings. Throughout the 30-day duration, the plant leaves were periodically shaken to ensure that the whiteflies moved among the plants. Before placing the plants in the cage and again after the 30 days, younger leaves from each test plant were collected to assess for CPMMV infection.

### 2.8. Statistical Analysis

Differences in seed-transmission frequencies were assessed using Fisher’s exact test for 2 × 2 contingency tables. Comparisons involving more than two groups were performed using the Fisher–Freeman–Halton exact test. When significant overall differences were detected, pairwise Fisher’s exact tests were conducted, and *p*-values were adjusted for multiple comparisons using the Bonferroni correction. Exact tests were preferred over Pearson’s chi-square tests because of the small sample sizes and the presence of expected cell counts below five.

## 3. Results and Discussion

### 3.1. CPMMV Is Seed-Transmitted in Common Beans

The first record of CPMMV in Brazil was in 1979, when it was observed in common bean plants showing angular mosaic symptoms [9]. Twenty years later, CPMMV reappeared in soybean fields in Goiás state, causing mosaic and stem necrosis symptoms. In the following years, CPMMV was found to be widely distributed in the country’s major soybean-producing areas [6,10,27]. Yield losses in soybeans have been experimentally estimated to range from 85% in older cultivars suffering from stem necrosis [10] to 14–16% in more recent asymptomatic cultivars [21]. Although CPMMV was considered non-significant in common beans when it was first reported [9], after about 35 years, by 2013, its presence had spread to eight Brazilian states [3], and its incidence reached as high as 100% in commercial fields in Central Brazil during the 2016 season [4].

Seed infection by a virus plays a key role in the disease epidemiology, as infected plantlets serve as primary inoculum sources early in the growing season. In addition, virus transmission through seeds enables the long-term persistence of viruses outside the host plant and facilitates their spread across continents. Serious epidemics can arise when the virus is transmitted by efficient vectors, such as whiteflies. Early studies found no evidence of seed transmission of CPMMV in soybeans or common beans in Brazil [9,10,19]. However, advancements in virus detection methods have enabled the detection of seed-transmitted viruses, even at low viral loads in the seedlings originating from infected seeds. In the past years, CPMMV has been frequently detected in bean-growing areas, even early in the season, despite low vector populations. This fact rekindled the concerns of CPMMV being seed-transmitted in beans.

**Figure 3:**
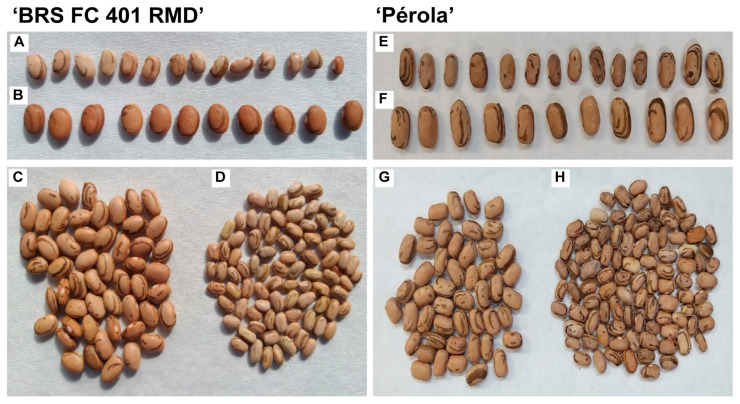
Common bean seeds of ‘BRS FC 401 RMD’ and ‘Pérola’ varieties used in seed transmission experiments. (**A**,**D**) ‘BRS FC 401 RMD’ seeds collected from bean plants in a field with viral symptoms observed in 80% plants. (**E**,**H**) ‘Pérola’ cultivar seeds were collected in a field with a high incidence of cowpea mild mottle virus (CPMMV), bean golden mosaic virus (BGMV), and bean-associated cytorhabdovirus (BaCV). (**B**,**C**,**F**,**G**) Seeds from plants grown in a greenhouse free of insects and tested negative for CPMMV through periodic sampling. The seed portions (**C**,**D**,**G**,**H**) from both cultivars are of equal weight. Photos by Leandro Ribeiro de Matos (‘BRS FC 401 RMD’) and Simone Ribeiro (‘Pérola’).

To tackle this issue, in Experiment I (Figure 1), we used seeds from the ‘BRS FC 401 RMD’ beans variety collected from a field with a high incidence of virus symptoms. The seeds collected from these plants were smaller, and some showed slight deformities compared to seeds from healthy plants (Figure 3). Plants emerging from these seeds appeared normal with no visual symptoms, corroborating a previous report on soybeans [16]. These authors suggested that the lack of symptoms in seedlings originating from CPMMV-infected plants might be due to host tolerance to early infection [16]. However, another study on soybeans showed that emerged seedlings from CPMMV-infected plants presented early symptoms, that intensified as the plants grew [13]. Since we had access to an anti-CPMMV antibody, and ELISA tests are commonly used to detect seed transmission of plant viruses, including CPMMV [14,28,29], we used PTA-ELISA as our first choice for CPMMV detection in this experiment. All 190 plants grown from field-collected seeds tested negative for CPMMV in the PTA-ELISA, whereas only the CPMMV-infected controls tested positive. Given that PCR-based techniques can detect viruses at picogram levels, making them more sensitive than ELISA, we also used RT-PCR to examine pooled samples of 10 plants each. All 19 bulked samples were negative for CPMMV, with amplicons of the expected size obtained only from the positive controls. These results suggested that either CPMMV is not transmitted through the seeds of this common bean cultivar or that CPMMV was present in very low concentrations in the plants and could not be detected by the methods used.

In Experiment II (Figure 1), we investigated the potential for low seed-transmitted CPMMV loads in plants using field-collected seeds of ‘BRS FC 401 RMD’ beans. We also included ‘Pérola’ seeds, which were similarly collected from field-grown plants. This time, all RT-PCR tests were conducted on individual plant samples. The seeds of ‘Pérola’ and ‘BRS FC 401 RMD’ collected from plants in a field with a high incidence of virus-like symptoms were visually smaller in size and mildly deformed (Figure 3). These observations contrast with another study, which found that CPMMV infection did not produce visible symptoms in soybean seeds [16]. In the present study, while ‘Pérola’ plants did not show any visual symptoms, some plants grown from CPMMV-infected ‘BRS FC 401 RMD’ seeds (which were from the same seed lot used in Experiment I) presented some deformed leaves, resembling CPMMV-infected plants.

**Figure 4:**
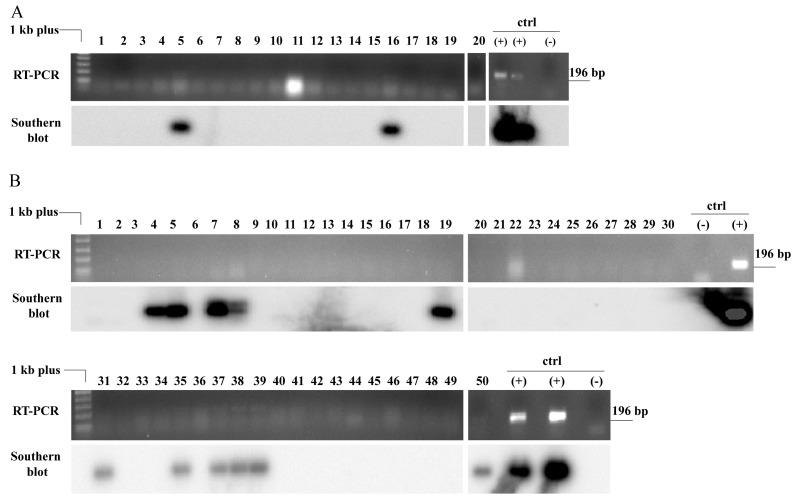
Cowpea mild mottle virus (CPMMV) can be transmitted by seeds in common beans. Bean seeds collected from plants in experimental fields with viral symptoms (‘BRS FC 401 RMD’) (**A**) and in a commercial field (‘Pérola’) (**B**) in which CPMMV was detected in high incidence were sown in a growth chamber. The plants were cultivated, and 30 days after germination, leaf samples were collected individually. Virus detection was performed by RT-PCR using specific primers (amplicon size 196 bp), followed by Southern blotting. In the gel of samples ‘BRS FC 401 RMD’ (**A**), very discrete bands are present in plants 5 and 16, while among the ‘Pérola’ cultivar (**B**), it is possible to notice bands in samples 35, 37, 38, and 39. Hybridization confirmed the infection in ‘BRS FC 401 RMD’ plants 5 and 16 and increased detection in ‘Pérola’ plants (4, 5, 7, 8, 19, 31, 35, 37,38, 39, and 50).

The RT-PCR reactions performed on RNA samples from plants grown from seeds (20 ‘BRS FC 401 RMD’ and 50 ‘Pérola’) produced very faint bands that matched the size (~200 nt) of the positive control plant infected with CPMMV. For the ‘BRS FC 401 RMD’ samples, a band was barely visible in two samples (Figure 4A, samples 5 and 16). In contrast, for the ‘Pérola’ plants, the band was detectable in four samples: #35, 37, 38, and 39 (Figure 4B). The positive controls yielded clear bands (Figure 4A,B). Given that equivalent amounts of RNA and cDNA from both the bean samples and the positive controls were used for the RT-PCR tests and considering the good quality of the cDNAs as assessed by PCR for the actin 11 gene, these results suggest that the CPMMV load in the plants grown from infected seeds was relatively low. The low levels of CPMMV in these samples could explain the negative ELISA and RT-PCR results for the bulk samples in Experiment I. Generally, RT-PCR assays are several orders of magnitude more sensitive than ELISA tests [29,30,31], especially in embryonic tissues compared to vegetative tissues [13]. Still, it was not possible to detect CPMMV-derived amplicons in bulked samples. While extracting RNA from bulk samples may save time and resources, the total RNA volume per sample can be lower in bulk processing than in individual samples, due to greater dilution.

Although CPMMV RT-PCR-derived amplicons were detected in some individual samples in Experiment II (Figure 1), they were at the limit of visual detection on the agarose gel. Therefore, we might have overlooked CPMMV in samples with low viral loads. Nucleic acid hybridization is a powerful technique for detecting low quantities of nucleic acids. It can detect viral nucleic acids in plants by Southern blotting, even when no visible virus-derived bands are apparent on the gels [32,33,34]. Hybridization with a virus-specific probe improves RT-PCR sensitivity by up to 100-fold. To determine whether there were additional positive samples with CPMMV-derived bands that were not visible on the gels, we blotted the agarose gels onto a nylon membrane and performed Southern blot hybridization using a CPMMV-specific probe. For the ‘BRS FC 401 RMD’ plants, the Southern blot hybridization results confirmed the RT-PCR, as a hybridization signal was detected only in samples 5 and 16 (Figure 4A). This indicates a 10% CPMMV seed transmission rate in this cultivar. Although some ‘BRS FC 401 RMD’ plants exhibited deformed leaves, this abnormality could not be linked to CPMMV infection. In the case of the ‘Pérola’ plants, Southern blot hybridization revealed seven additional infected plants (#4, 5, 7, 8, 19, 31, and 50) in addition to the four already identified by RT-PCR (Figure 4B). This totalized 22% CPMMV seed transmission in the ‘Pérola’ cultivar. The variation in seed transmission rates among cultivars of the same plant species has been observed before for CPMMV and several other viruses, including barley stripe mosaic virus—BSMV (*Hordeivirus hordei*, family *Virgaviridae*), pea seed-transmitted mosaic virus—PSbMV (*Potyvirus pisumsemenportati*, family *Potyviridae*), alfalfa mosaic virus—AMV (*Alfamovirus AMV*, family *Bromoviridae*), soybean mosaic virus—SMV (*Potyvirus glycitessellati*, family *Potyviridae*), and tomato yellow leaf curl virus—TYLCV (*Begomovirus coheni*, family *Geminiviridae*) [8,35,36,37,38,39,40].

Together, the results from Experiment II (Figure 1) demonstrate that CPMMV from Brazil is transmitted through common bean seeds of two cultivars, ‘BRS FC 401 RMD’ and ‘Pérola’. Also, it was crucial to combine RT-PCR and Southern blot hybridization to achieve sufficient sensitivity to reliably detect CPMMV seed transmission in the bean plants. Accordingly, RT-PCR followed by Southern hybridization was used to detect CPMMV-infected plants in all the other experiments.

The level of virus symptoms in plants from the fields where the samples from experiments I and II were collected exceeded 80%. However, we did not test the mother plants for CPMMV infection. Thus, we cannot be sure if all the analyzed plants were grown from seeds derived from CPMMV-infected mother plants. To assess whether seed transmission rates differ when seeds are derived from mother plants unequivocally infected with CPMMV, we conducted experiments III and IV.

**Figure 5:**
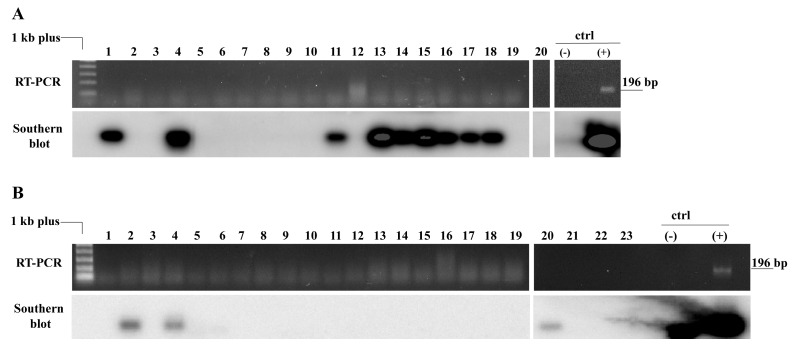
Cowpea mild mottle virus (CPMMV) can be transmitted by seeds to the progeny of mechanically inoculated ‘Pérola’ and ‘BRS FC 401 RMD’ common bean plants. After mechanical inoculation with CPMMV, the seeds of infected plants were collected in a pool. Seeds were sown, and the plants were maintained in a growth chamber until virus detection 30 days after germination (dag). Virus detection was performed by RT-PCR, using specific primers (amplicon size 196 bp), followed by Southern blotting. (**A**) Nine ‘BRS FC 401 RMD’ (1, 4, 11, 13, 14, 15, 16, 17, 18) and (**B**) three ‘Pérola’ (2, 4, 20) were positive for CPMMV.

RT-PCR and Southern blot analysis of the plants grown from seeds collected from CPMMV-infected mother plants that were inoculated by mechanical means in experiment III resulted in 45% of CPMMV transmission through seeds for ‘BRS FC 401 RMD’ and 13% for ‘Pérola’ (Figure 5). Other authors have also confirmed the transmission of CPMMV to plant progeny via seeds obtained from mechanically infected plants [13]. However, the seed transmission rate of CPMMV was higher in soybean grow-out tests, reaching 86–100% in some studies across different countries [13,16]. It should be emphasized that the presence of a virus in seeds does not always imply its transmission to the offspring, as the virus could be localized in the endosperm rather than in the embryo [41]; therefore, grow-out assays remain the most reliable method for determining seed transmission of viruses.

**Figure 6:**
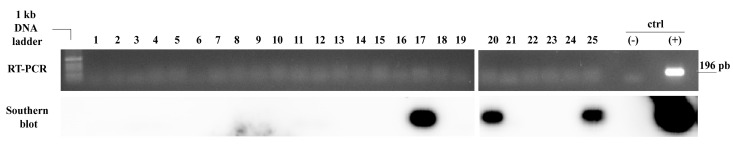
Cowpea mild mottle virus (CPMMV) can be transmitted by seeds to the progeny of whitefly-inoculated ‘BRS FC 401 RMD’ common bean plants. Twenty-five seeds of plants inoculated with CPMMV and BaCV by the whitefly *Bemisia tabaci* (MEAM1) were sown and maintained in a growth chamber until RNA extraction, 30 days after germination. CPMMV was detected by Southern blot in three plants (17, 20, and 25).

**Figure 7:**
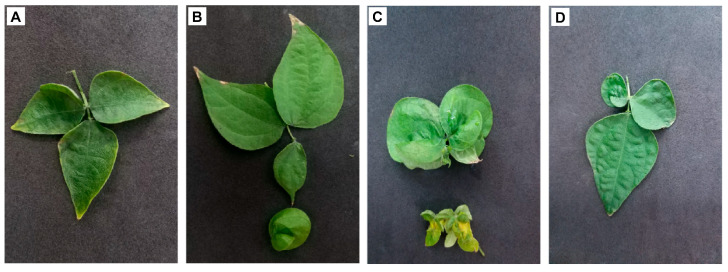
Leaves of common bean ‘BRS FC 401 RMD’ grown from seeds of mother plants inoculated with CPMMV by whitefly (*Bemisia tabaci* MEAM1). (**A**,**B**) CPMMV-positive plants and (**C**,**D**) CPMMV-negative plants in Southern blot hybridization.

In an identical analysis of ‘BRS FC 401 RMD’ bean plants obtained from seeds of CPMMV-infected mother plants that were inoculated by the whitefly vector in Experiment IV, a rate of 12% CPMMV seed transmission was achieved (Figure 6). Some of these plants showed leaf deformations, although it was not possible to correlate this fact with the presence of CPMMV. For example, leaf deformations were observed in both positive samples 20 and 25 (Figure 7A,B) and in negative samples such as 1 and 2 (Figure 7C,D).

Seed-transmission rates between cultivars were compared using Fisher’s exact test. No significant difference was observed between Pérola and BRS-FC401 RMD in Experiment II (*p* = 0.322) (Table 1), which also represents different seed sources (commercial field for Pérola and experimental field for BRS FC 401 RMD). In contrast, Experiment III revealed a significantly higher seed-transmission frequency in BRS-FC401 RMD (9/20 plants) than in Pérola (3/23 plants) (Fisher’s exact test, two-tailed, *p* = 0.039). Seed-transmission frequencies in the cultivar BRS-FC401 RMD differed significantly among experiments (Fisher–Freeman–Halton exact test, *p* = 0.0177). Pairwise Fisher’s exact tests revealed that Experiment III showed a higher transmission rate (9/20; 45.0%) than Experiments II (2/20; 10.0%; *p* = 0.029) and IV (3/25; 12.0%; *p* = 0.024), whereas no difference was detected between Experiments II and IV (*p* = 1.000). However, these differences were no longer significant after Bonferroni adjustment for multiple testing (adjusted α = 0.0167). Thus, the overall analysis supports variation among experiments, but no individual pairwise contrast was statistically significant after correction for multiple comparisons.

The results for ‘BRS FC 401 RMD’ could be linked to the fact that the mother plant was undoubtedly infected with CPMMV. However, several abiotic factors such as temperature [42,43], drought [44], and light intensity [43,45], as well as biotic factors like the virus isolate [46,47], host genotype [8,12,16,46,48], and phenological stage at which the plant became infected [49], may also influence the production of virus-infected seeds. A recent study has shown that an antiviral RNAi pathway, which is more strongly expressed in seeds than in whole plants, can inhibit the seed transmission of viruses in plants [50]. However, there is limited knowledge of proteins with silencing-suppressor functions encoded by the CPMMV genome. Some studies suggest that the CRP protein, encoded by ORF6 of carlaviruses, may play a role in suppressing silencing [51,52,53]. In fact, RNAi-mediated inhibition of seed transmission has been demonstrated only in the cucumber mosaic virus (CMV)-*Arabidopsis thaliana* model system to date [50]. As a genetically defined mechanism, RNAi-mediated suppression of vertical virus transmission may be hypothetically influenced by both host and virus genotypes, which could help explain the observed variation in transmission rates. Moreover, evidence indicates that seed transmission exerts selective pressure toward decreased virulence in plant viruses while enhancing host resistance [54]. This phenomenon could account for the variation in seed transmission rates observed among different cultivars.

The source plants that provided the seeds analyzed in Experiments I and II were not tested for the presence of CPMMV. This implies that some seeds may have originated from uninfected plants, which could have influenced the observed seed transmission rates. However, we emphasize that cultivar BRS FC 401 RMD is immune to BGMV, suggesting that the viral symptoms observed in these plants (and in their seeds, which were smaller and malformed) were likely caused by CPMMV. Furthermore, the seed transmission rates obtained in these experiments reflect natural occurrence in seeds harvested from a field exhibiting viral symptoms, considering the variable infection status among plants. Additionally, the mechanical inoculation did not result in a significantly higher seed transmission rate, as stated before.

Taken together, our findings show that the seed transmission rates of CPMMV for the ‘BRS FC 401 RMD’ common bean variety range from 10% to 45%, while for the ‘Pérola’ variety, the rates range from 13% to 22% (Table 1). These rates are somewhat higher than those recorded for common bean cultivars infected with the CPMMV isolate from Ghana [8]. Additionally, the transmission rates reported in the present study are at least 26.6 times higher than those reported for the BMX POTÊNCIA RR soybean cultivar in Brazil [21], but lower than those observed in soybean in other countries [13,16], suggesting that host-specific factors may affect seed transmission efficiency.

### 3.2. No Evidence Was Found for the Seed Transmission of BaCV

Plant rhabdoviruses are generally not considered to be seed transmitted [55,56]. However, since the ‘BRS FC 401 RMD’ and ‘Pérola’ seeds were collected from bean plants in regions where BaCV is prevalent [4], and there is no data on BaCV seed transmission, we applied the same detection method used for CPMMV to investigate BaCV. Following RT-PCR with BaCV primers and Southern hybridization using a BaCV-specific radiolabeled probe, all tested plants had negative results, except for the positive control. This indicates that, at least in these plants and under the experimental conditions, BaCV was not transmitted via seeds. The seed transmission of papaya virus E (PpVE), another *Betacytorhabdovirus caricae* isolate found in *Carica papaya* with a genome sequence identity of 97% to BaCV, has been evaluated by Medina-Salguero et al. [57]. Grow-out tests from 100 seeds obtained from PpVE-positive papaya and *Macroptilium lathyroides* plants were conducted, and similar to the findings for BaCV in common beans, this mode of transmission was not observed [58].

### 3.3. Seed-Transmitted CPMMV Could Be an Inoculum Source in Secondary Transmission

**Figure 8:**
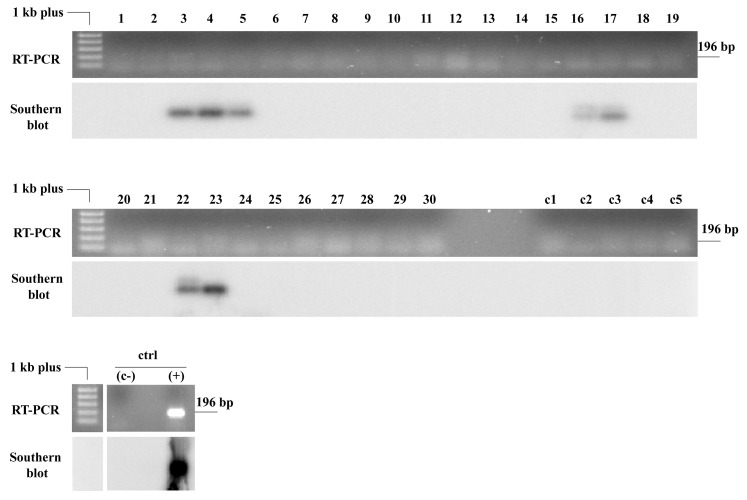
Cowpea mild mottle virus (CPMMV) seed-transmitted in common bean is an inoculum source for secondary transmission by the whitefly *Bemisia tabaci* (MEAM1). Three CPMMV-positive ‘BRS FC 401 RMD’ plants obtained in a seed transmission grow-out assay were introduced into three BugDorm cages with whiteflies and maintained for 48 h. Ten seedlings of the same cultivar were introduced into each cage (cage 1: plants 1–10; cage 2: plants 11–20, and cage 3: plants 21–30) and maintained for 30 days. As a control, five bean plants (c1–c5) were introduced into a fourth cage containing only whiteflies. At the end of this period, the last newly formed leaf of each bean plant was collected and analyzed by RT-PCR and Southern blot. In the gel, faint bands are visible in samples 3, 22, and 23 at the expected size of 196 bp. In the Southern blot, samples 3, 4, 5, 16, 17, 22, and 23 were positive. The five controls c1–c5 were negative. C+ is the positive control.

Our results from the whitefly transmission assay (Experiment IV, Figure 1) confirmed that vertically transmitted CPMMV in the common bean ‘BRS FC 401 RMD’ can potentially act as an inoculum source for secondary infections, at least under experimental conditions in a growth chamber. CPMMV was detected using RT-PCR followed by Southern blot analysis 30 days after inoculation in 7 out of 30 test plants, representing approximately 23% of the samples (Figure 8). CPMMV was not detected in any of the five plants that served as controls for the experiment (Figure 8—c1 to c5). Additionally, leaf samples collected from all test plants before the transmission assay tested negative for the virus. The results indicate that although the viral load in the progeny of infected bean plants appears to be low, these plants can still act as a source of inoculum for new CPMMV infections in healthy beans, particularly via the whitefly vector, *B. tabaci* MEAM1. A vector-mediated transmission rate of 23.3% underscores the significance of seed transmission within the crop, as these infected plants will be a primary source of CPMMV in common bean fields. If an efficient vector, such as whiteflies, is present in this context, yield losses could be substantial, even if virus transmission through seeds occurs at low rates [59]. Taken together, the results suggest that the virus can spread in the field when CPMMV-infected seeds are sown, which act as a source of inoculum for vector transmission.

The occurrence of CPMMV in common bean crops has been highlighted since 2013 [3], with high incidence rates reaching up to 94–95% of tested samples in some Brazilian states [4]. Our results suggest that the whitefly may not be the only factor contributing to these high rates. The impact of CPMMV on common beans is not well understood, underscoring the need for further research. However, given its seed-transmitted nature—where transmission rates can be as high as 45%—and the reductions in soybean productivity associated with the virus [21], it is reasonable to anticipate that CPMMV could pose a significant threat to bean production. Hence, the development and implementation of preventive and management strategies may become increasingly relevant for this crop in the future. The establishment of certified seed fields with strict whitefly control could serve as a viable solution to minimize primary infections during sowing.

## Figures and Tables

**Table 1:** Cowpea mild mottle virus (CPMMV) seed transmission in common beans in Brazil.

Number of Positive Plants/Number of Tested Plants (Seed Transmission %)			
Cultivar	Experiment II ^1,2^	Experiment III ^3^	Experiment IV ^4^
Pérola	11/50 ^1^ (22%) Aa	3/23 (13%) Aa	-
BRS FC 401 RMD	2/20 ^2^ (10%) Aa	9/20 (45%) Bb	3/25 (12%) a
Total	13/70 (18.6%) a	12/43 (27.9%) a	3/25 a

Seeds from commercial field ^1^, experimental field ^2^, mechanical inoculation assay ^3^, and whitefly-mediated inoculation assay ^4^. Means followed by different uppercase letters within columns or lowercase letters within rows are statistically different at *p* < 0.05 by the Fisher’s exact test. For the cultivar BRS FC 401 RMD, pairwise Fisher’s exact tests revealed that Experiment III showed a higher transmission rate (9/20; 45.0%) than Experiments II (2/20; 10.0%; *p* = 0.029) and IV (3/25; 12.0%; *p* = 0.024), whereas no difference was detected between Experiments II and IV (*p* = 1.000). However, these differences were no longer significant after Bonferroni adjustment for multiple testing (adjusted α = 0.0167).

## Data Availability

Data are contained within the article.

## Supplementary Materials

The following supporting information can be downloaded at: https://www.mdpi.com/article/10.3390/v18070752/s1, Table S1: Primers used for quality test and for virus detection in common bean plantlets by RT-PCR.
