# Supplementary material for: Seed Transmission of Cowpea Mild Mottle Virus in Common Beans in Brazil
Source: Viruses. 2026 Jul 7;18(7):752. doi: 10.3390/v18070752 (PMC13431507; doi:10.3390/v18070752)
Supplement: Supplementary file 1 [file viruses-18-00752-s001.zip › viruses-4380719-supplementary.pdf]

Table S1. Primers used for quality test and for virus detection in common bean plantlets by RT-PCR.

Target	Primer name	Primer sequence 5’- 3’	Amplicon size (bp)	Tm (°C)	Reference
**CPMMV**	qCPMMV_ 4144 F	GAAGGGTTTCCATCCAAGGT	196	58	This study
	qCPMMV_ 4339 R	TTGTTCGAGTTCCCTCCATC			
**CPMMV**	CPMMV-4000F	AACTTGGCCTTAGTGAACTCTACA	500	58	[3]
	CPMMV-4500R	ATTAGCTCTGTGCCTGGGGT			
**BaCV**	BaC_1F	GTTCAAAACTTTATAACCGCAGGAG	1579	61	[4]
	BaC_1579R	CGCAACAGATTAAACAGGAAAT			
**Actin 11**	qAct11_F	TGCATACGTTGGTGATGAGG	150	58	[26]
	qAct11_R	AGCCTTGGGGTTAAGAGGAG			
